# A Review of Natural Products for Prevention of Acute Kidney Injury

**DOI:** 10.3390/medicina57111266

**Published:** 2021-11-18

**Authors:** Hyun Goo Kang, Hyun Ki Lee, Kyu Bong Cho, Sang Il Park

**Affiliations:** 1 Department of Optometry, Catholic Kwandong University, Gangneung 20561, Korea; hgkang@cku.ac.kr; 2 School of Game, DongYang University, Dongducheon 11307, Korea; hyunki@dyu.ac.kr; 3 Department of Biomedical Laboratory Science, Shinhan University, Uijeonbu 11644, Korea; kbcho@shinhan.ac.kr

**Keywords:** acute kidney injury, prevention, natural products, antioxidant

## Abstract

Background and Objectives: acute kidney injury (AKI), formerly called acute renal failure (ARF), is commonly defined as an abrupt decline in renal function, clinically manifesting as a reversible acute increase in nitrogen waste products—measured by blood urea nitrogen (BUN) and serum creatinine levels—over the course of hours to weeks. AKI occurs in about 20% of all hospitalized patients and is more common in the elderly. Therefore, it is necessary to prevent the occurrence of AKI, and to detect and treat early, since it is known that a prolonged period of kidney injury increases cardiovascular complications and the risk of death. Despite advances in modern medicine, there are no consistent treatment strategies for preventing the progression to chronic kidney disease. Through many studies, the safety and efficacy of natural products have been proven, and based on this, the time and cost required for new drug development can be reduced. In addition, research results on natural products are highly anticipated in the prevention and treatment of various diseases. In relation to AKI, many papers have reported that many natural products can prevent and treat AKI. Conclusions: in this paper, the results of studies on natural products related to AKI were found and summarized, and the mechanism by which the efficacy of AKI was demonstrated was reviewed. Many natural products show that AKI can be prevented and treated, suggesting that these natural products can help to develop new drugs. In addition, we may be helpful to elucidate additional mechanisms and meta-analysis in future natural product studies.

## 1. Introduction

Acute kidney injury (AKI) refers to a sudden decrease in renal function and decreased renal function is characterized by an increase in serum creatinine (sCr) levels and an abnormal decrease in urine output [1]. AKI, previously called acute renal failure (ARF), is a condition of sudden kidney failure in patients with or without preexisting chronic kidney disease (CKD); severe kidney dysfunction within a few hours or days results in a significant decrease (oliguria) or complete elimination of urine (anuria), with electrolyte imbalance, often requiring hemodialysis [2]. In AKI’s population-based study using the risk, injury, failure, loss, end-stage kidney disease (RIFLE) criteria, the annual incidence of AKI was 2147 per million people. In another collaborative study, the annual incidence of non-dialysis and dialysis-dependent AKI was 3841 and 244 per million people, respectively [3]. In addition, if not treated immediately, AKI can lead to development of CKD overtime requiring replacement therapies such as dialysis and in the best-case kidney transplantation [4]. It is known that approximately 20% of patients with a history of AKI develop a chronic disease characterized by cardiovascular complications, and increased mortality. Various pathological mechanisms have been suggested that progression AKI and transition to CKD including anoxemia, thinning of microcirculation vessels, altered phenotype and function of cells residing in the kidneys, G2/M phase cell cycle arrest, continuous chronic inflammation, development of epileptic fibrosis, mitochondrial fragmentation, epigenetic modifications, activation of renin-angiotensin system, cell and tissue senescence [5].

Basically, AKI is a term that describes a clinical disease that occurs when kidney function is severely impaired, waste builds up in the body, and electrolytes, the acid-base balance, and water are out of balance [6]. AKI can significantly increase morbidity and mortality. It is also common in hospitalized patients and increases the risk of CKD and end stage renal disease (ESRD) [6]. AKI occurs in 5–10% of all hospitalized patients and is reported in 60% of intensive care unit (ICU) patients [7]. According to recent studies have shown that sepsis and hypovolemia are the most common causes of AKI in critically ill patients, followed by nephrotoxic substances [8].

The definition and staging of AKI are based on the criteria RIFLE, and previously defined criteria of the Network for Acute Kidney Injury (AKIN). In the clinical practice guidelines on Kidney Disease Improving Global Outcome (KDIGO), AKI is defined as one of the following: sCr increases by 0.3 mg/dL or more (26.5 μmol/l or more) within 48 h. Or when sCr is increased more than 1.5 times baseline. This is known or estimated to have happened within the last a week, Or urine volume < 0.5 mL/kg/h for 6 h [3].

Cellular and molecular targets are relevant to the pathogenesis of AKI, such as damage in the plasma membrane, gene expression, alterations in the actin cytoskeleton, stress due to accumulation of unfolded proteins on the endoplasmic reticulum, swelling with rarefaction of the cristae, and mitochondrial fragmentation, cell-surface receptors in both initiation and/or propagation of epithelial injury, cell proliferation limitation, lysosomal disruption [9].

Due to the lack of established therapeutic interventions for AKI, patients with AKI can only rely on prophylaxis and early diagnosis of AKI to reduce adverse effects and mortality [10]. Kashani K et al. suggested that the scope of AKI treatment should range from risk assessment and prevention in the community to the prevention of AKI in hospitals, the optimization of AKI treatment, and ultimately the prevention of AKI recurrence [11]. However, effective treatments are still deficient, because oxidative stress, inflammation, damage, and repair imbalances in kidney disease are deeply involved in the pathological process of specific AKI targets [12].

Natural products have long been used to treat and prevent various disease [13]. Several natural compounds in natural products have demonstrated good effects and high efficacy in suppressing cell death, oxidative stress, and inflammation [12]. In several studies, in order to examine the efficacy of these natural products, the efficacy of prevention and treatment for AKI was been confirmed and suggested in in vivo and in vitro tests [14].

The purpose of this study was to comprehensively investigate and summarize the mechanism by which natural products exhibit efficacy in relation to AKI in vivo and in vitro.

## 2. Research Method

In order to find research results on AKI and natural products, many papers published between 2010 and 2021 were searched for in electronic databases such as PubMed, Google Scholar, and Embase. We identified articles for further review in compounds by performing an initial screen of identified abstracts or titles about prevention against AKI. Papers were considered for inclusion if they were researched on anti-oxidant and anti-inflammatory effects. The keywords for the search were as follows: “acute kidney injury”, “AKI”, “natural product”, “compounds”, “plant”, and “antioxidant”. The papers finally selected for the literature review are shown in Table 1.

## 3. Pathophysiology of AKI

**Figure 1:**
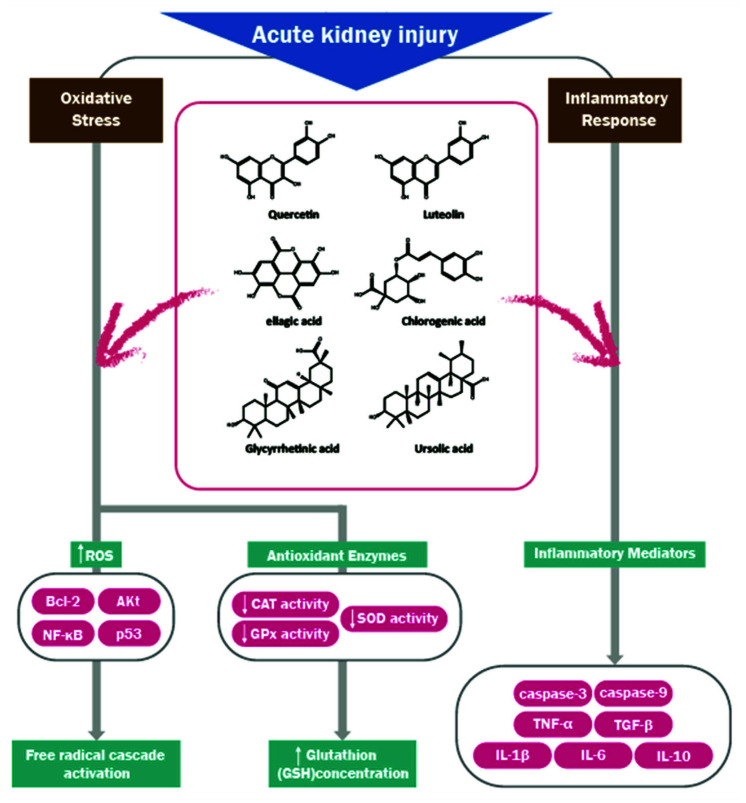
Oxidative stress and inflammatory response mechanisms involved in the pathogenesis of aucte kidney injury and some effect compounds. Abbreviation ROS; Reactive oxygen species, CAT; catalase, GPX; Glutathione peroxidase.

AKI is a clinical endpoint of many processes that lead to a decreased glomerular filtration rate and are indicators of general kidney function. Key components of the injury process include apoptosis, necrosis, reactive oxygen species (ROS), and microvascular injuries that cause local ischemia, endothelial dysfunction, leakage, and inflammation (Figure 1) [45]. AKI is most commonly caused by ischemic or toxic damage and occurs in septic situations. Components of the pathophysiology are inflammatory reactions as well as tubular or vascular damage and their consequences [46].

Inflammation is mediated in part by the adhesion of leukocytes to diseased endothelial cells. Ischemic AKI is the most common cause of AKI in hospitalized patients, with an average mortality rate of 50% [47]. In response to injury, surface expression of the leukocyte adhesion molecules ICAM-1 and P and E selectins is increased on endothelial cells [47,48,49]. In vivo imaging studies have shown that leukocytes adhere to the wall of peritubular capillaries within hours of reperfusion [50]. Treatment aimed at reducing endothelial/white blood cell interactions by targeting these endothelial adhesion molecules can maintain blood flow and prevent ischemia reperfusion induced nephropathy [47]. Endothelial cells can also be a source of chemokines such as fractalkine (CX3CL1), which is expressed after kidney injury, and can promote are infiltration of macrophages [47].

## 4. Natural Products for the Prevention of AKI

Since AKI is a multifactorial disease and can be associated with co-morbidities, there is no pharmacological approach in clinic to reverse the renal injury. Currently, maintenance of volume homeostasis and correction of biochemical abnormalities are still the goals for the treatment of AKI. Therefore, prevention is always critical for this disease. Some functional components from food materials have been reported to have the ability to protect renal functions, indicating long-term administration of these components might be an effective approach to prevent AKI [51].

As shown in Figure 1, the TGF-β receptor, TNF receptor, caspase-3, caspase-9, etc., then these receptors activate downstream pathways, further ROS production and inflammatory responses, eventually leading to kidney damage. Several effective natural products suppress cisplatin, lipopolysaccharide (LPS), Ischemia-Reperfusion (I/R)-stimulated inhibiting the NF-κB pathway and reducing inflammation. Moreover, some compounds reduce apoptosis by inhibiting TGF-β, Akt, and p53 pathways. Additionally, some compounds can reduce ROS production.

Many compounds, including flavonoids, polyphenols, terpenes, alkaloids, saponins, and quinones, have multiple beneficial pharmacological activities such as antioxidants and anti-inflammatories [52]. Oxidative stress is considered an AKI factor. Natural products derived from plants have strong anti-inflammatory and antioxidant properties. There have been many studies to investigating the effect of common herbal extracts and their constituents on AKI [53]. This section discusses extracts of several plants and isolated compounds used for the prevention and treatment of AKI.

### 4.1. Flavonoids

Flavonoids are found in many plant foods, including vegetables, fruits, and herbs. The flavonoid activity depends on the structure of the hydroxylated phenol [54]. Flavonoids are known to have anti-cancer, anti-inflammatory, and antioxidant effects [55,56,57].

Quercetin is a naturally occurring flavonoid compound commonly found in more than 20 fruits and vegetables and is the most abundant in the diet. It has been of medical interest because it is known for its pharmacological effects such as anti-inflammatory, antihypertensive, vasodilator, anticholinergic, and anti-atherosclerosis treatment [58]. Y. Wang et al. reported that quercetin inhibited ferroptosis in renal proximal tubular epithelial cells. This compound blocked the typical morphologic changes of ferroptotic cells by reducing the levels of malondialdehyde (MDA) and lipid ROS and increasing glutathione levels [15].

Luteolin is a flavonoid component found not only in peanut shells, but also in parsley, celery, pepper, and chamomile, and is known to have anticancer, anti-inflammatory, and antioxidant effects [59]. Treatment with luteolin in mice treated with cisplatin can significantly improve renal dysfunction and reduce renal tubular cell damage, oxidative stress, and apoptosis [16].

Apigenin is found in herbs such as thyme and parsley, and in orange, peppers. It can improve I/R damage to the heart, brain and liver of rats, as well as epithelial cells of the proximal tubules of the human kidney in vitro. medicine. It can also reduce induced nephrotoxicity [60]. X. Wang et al. reported that apigenin significantly up-regulates the expression of B-cell lymphoma 2 (Bcl-2) and phosphor-Akt (p-Akt), Phosphoinositide 3-kinase (PI3K), while down-regulating the expressions of Caspase3 and Bax induced by hypoxia/reoxygenation injury [17].

Kaempferol is a natural dietary flavonoid compound with many adaptive biological activities, including antioxidant and estrogenic activity. Z. Wang et al. evaluated the effect of kaempferol on mechanisms related to nephrotoxicity in a cisplatin-induced AKI mouse model. Pretreatment with kaempferol has been observed to reduce kidney damage [18].

Icariin is the main active flavonolic glycoside of the epimedium. It is widely used in medical treatment due to its anti-tumor properties and potential for and osteoporosis treatment and has been shown to slow cell aging [61,62]. Icariin may improve urinary protein excretion and renal tissue damage in pregnancy induced hypertension rats, and its main mechanism is mediated in part by the up-regulation of nephrin expression and down-regulation of Ang II [19].

Myricetin is commonly found in fruits, berries, and vegetables. It has been shown to have a variety of biological activities, including antioxidant and anti-inflammatory effects [63]. Milicetin significantly increases GSH level and caspase activity, and at the same time improves renal tissue histopathology and significantly decreases levels of blood urea nitrogen (BUN), serum creatinine, caspase-3, TNF-α, IL-6, COXI, COXII and MDA. This study suggests a protective and promising prophylactic strategy to prevent nephrotoxicity [20].

**Figure 2:**
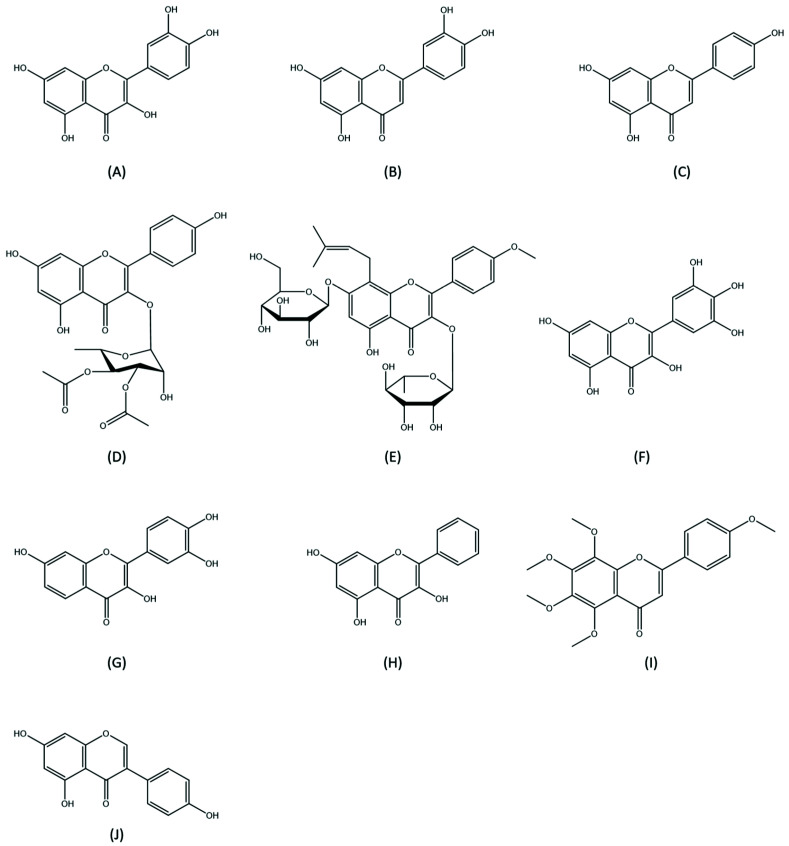
Chemical structures of flavonoids with potential preventive effects of aucte kidney injury. (**A**) Quercetin; (**B**) Luteolin; (**C**) Apigenin; (**D**) Kaempferol; (**E**) Icariin; (**F**) Myricetin; (**G**) Fisetin; (**H**) Galangin; (**I**) Tangeretin; (**J**) Genistein.

Other flavonoids including genistein, hesperetin, galangin, and fisetin, also prevent AKI—they are listed in Table 1 [21,22,23,24] and their chemical structures are shown in Figure 2.

### 4.2. Polyphenols

Polyphenols are found in vegetables, fruits, grains, chocolate, and beverages such as wine, coffee, black tea, and green tea [64]. Growing research shows that polyphenols may play an important role in health by regulating body weight, chronic disease, cell proliferation, and metabolism [65]. Epidemiological studies in humans and animals have shown that various polyphenols have anti-inflammatory and antioxidant properties, as well as therapeutic and prophylactic effects in obesity, cancer, cardiovascular, and neurodegenerative diseases [66,67,68,69].

Ellagic acid is an antioxidant and anti-inflammatory polyphenol compound found in tea, berries, nuts, and grapes [70,71,72]. Neamatallah et al. reported that ellagic acid nano improved the histopathological changes induced by cisplatin, such as tubular dilatation, necrosis, and degeneration [25].

Chlorogenic acid is one of the most readily available phenolic compounds in coffee, tea and other foods, and a well-known antioxidant [73]. This compound dose-dependently attenuated LPS-induced kidney histopathologic changes, serum BUN, and creatinine levels, and also suppressed LPS-induced TNF-α, IL-6, and IL-1β production both in serum and renal tissues [26].

Gallic acid is a low-molecular weight triphenol compound that has been shown to have strong antioxidant activity in many studies [74,75,76]. It provides effective protection against oxidative damage caused by active substances commonly found in biological systems [77,78]. Ahmed Vander H et al. reported that pretreatment with gallic acid can significantly increase levels of renal MDA, serum glutathione, and glutathione peroxidase activity after renal ischemia-reperfusion injury [27].

Vanillic acid is a phenolic derivative obtained from edible plants and fruits with antibacterial, antifilarial and hepatoprotective properties [79,80]. Due to the presence of carboxyl groups, vanillic acid is an important antioxidant and inhibits inflammatory mediators, inhibiting NF-κB in mice stimulated with LPS [79]. Sindhu G et al. suggested that pretreatment with vanillic acid (50 and 100 mg/kg) restored elevated levels of kidney function markers and reduced antioxidant status to near normal when compared to mice treated with cisplatin alone [28].

Resveratrol is a polyphenolic substance that is produced when plants are exposed to adverse environmental conditions such as fungi and pests. It is derived from a variety of edible plants such as grapes, berries and peanuts [81]. Its anti-inflammatory effect may prevent AKI caused by sepsis [82]. Resveratrol significantly ameliorated serum creatinine.

BUN, and histopathological lesions induced by cisplatin. In addition, it leads to significantly increased expression of Fas ligand, tumor necrosis factor-α (TNF-α), caspase-8 and Bcl-2 associated protein X apoptosis regulator (Bax), and decreased expression of anti-apoptosis regulators. Resveratrol administration significantly altered the cisplatin-induced changes in proteins associated with apoptosis [29].

Anthocyanins are water-soluble pigments that can be red, purple, blue or black depending on environmental pressure, such as solar radiation and low nitrogen content [83]. Anthocyanins contribute significantly to the antioxidant properties of some colored foods such as grapes and berries [84]. Li L. et al. showed that anthocyanins are effective against AKI by reducing inflammation, oxidative stress, lipid peroxidation and apoptosis [30].

**Figure 3:**
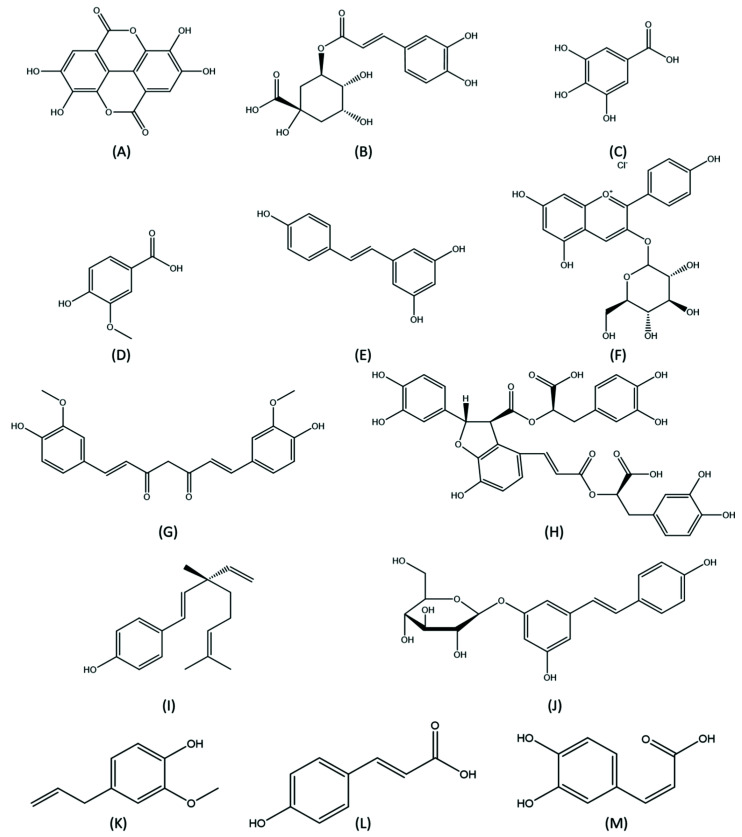
Chemical structures of polyphenols with potential preventive effects for aucte kidney injury. (**A**) Ellagic acid; (**B**) Chlorogenic acid; (**C**) Gallic acid; (**D**) Vanillic acid; (**E**) Resveratrol; (**F**) Anthocyanin; (**G**) Curcumin; (**H**) Salvianolic Acid B; (**I**) Bakuchiol; (**J**) Polydatin; (**K**) Eugenol; (**L**) *p*-Coumaric acid; (**M**) Caffeic acid.

Other flavonoids such as curcumin, salicylic acid B, bakuchiol, polydatin, eugenol, *p*-coumaric acid and caffeic acid also have noticeable effects [31,32,33,34,35,36,37,85,86], and are listed in Table 1, while their chemical structures are shown in Figure 3.

### 4.3. Terpenoids

Terpenoids, also known as isoprenoids, are the largest type of secondary metabolites in plants, accounting for about 60% of phytochemicals [87]. They have a distinctive fragrance and are used in spices and in traditional pharmaceuticals for perspiration, antipyretic, and analgesic effects [88,89]. Terpenoids are also used for cancer treatment and prevention, cardioprotection, endocrinology/reproductive dysfunction, nutritional supplements, conventional medicine, immunology, anti-inflammation, menopause, and neuroprotection [90].

Glycyrrhetinic acid is an effective ingredient of *Glycyrrhiza glabra* L. (Liquorice). It is very sweet and is used extensively as a conditioner and flavoring agent to treat a variety of inflammatory conditions [91]. Sana M et al. reported that glycyrrhetinic acid has a protective effect on methotrexate-induced nephrotoxicity and the possible mechanisms for activating the Nrf2/ARE signaling pathway to reduce oxidative stress and inflammation [38].

Ursolic acid (UA) is a naturally occurring triterpene compound found in various plants such as fruits and vegetables. Ursolic acid has been studied for its beneficial effects, such as anti-inflammatory, antioxidant, anti-apoptotic, and anti-cancer effects [92]. Recently, it was demonstrated that ursolic acid can treat sepsis in animal models [93]. According to a recent study, ursolic acid can protect against AKI-induced sepsis by inhibiting ROS and inflammatory cytokines, including TNF-α, IL-1β, and IL-6, in the kidneys of septic mice [39].

**Figure 4:**
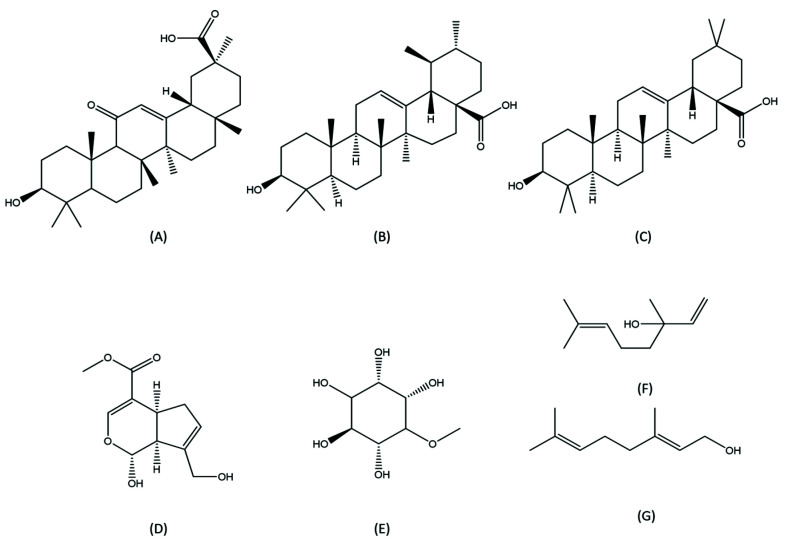
Chemical structures of terpenoids with potential preventive effects of aucte kidney injury. (**A**) Glycyrrhetinic acid; (**B**) Ursolic acid; (**C**) Oleanolic acid; (**D**) Genipin; (**E**) Pinitol; (**F**) Linalool; (**G**) Geraniol.

Oleanolic acid is a pentacyclic triterpenoid compound that is found in plant of the Oleaceae family such as the olive plant [94]. It is a natural product isolated from some food and medicinal plants. It is a triterpenoid that has many health benefits including antioxidant, anti-inflammatory, and anti-apoptotic effects [95]. Oleanolic acid inhibited the increase in proapoptotic caspase-3 and -9 activation and a simultaneous increase in poly (ADP-ribose) polymerase (PARP) cleavage activation in a concentration-dependent manner [40]. Genipin, pinitol, linalool, geraniol, malbiin, betulinic acid, butyric acid, and corosolic acid can also be used to prevent AKI and listed in Table 1 [41,42,43,44], while their chemical structures are shown in Figure 4.

## 5. Discussion

In this paper, 30 papers related to the prevention and treatment of AKI through natural products were reviewed. The AKI animal model was induced by drugs by cisplatin, LPS, Methotrexate, contrast, and glycerol. In addition, various induced-AKI models were tested, such as I/R injury, pregnancy, pancreatitis, and sepsis. Among the induced AKI models, 14 cases of cisplatin, 5 cases of I/R injury, LPS, and sepsis, 1 case of contrast, methotrexate, pregnancy-induced hypertension, glycerol, pancreatitis were tested. The main clinical features for evaluating renal function in AKI are an increase in sCr levels and BUN, and a decrease in urine output. In this case, 30 natural products tested in vivo showed the effect of reducing sCr levels and BUN, and this mechanism appeared was confirmed through various pathways. These pathways were involved in apoptosis, anti-oxidant, and inflammation.

We reviewed effective natural products against AKI by dividing them into flavonoids, polyphenols, and terpenoids according to their structural characteristics; flavonoids (quercetin, luteolin, apigenin, kaempferol, icariin, myricetin, fisetin, galangin, tangeretin, and genistein), polyphenols (ellagic acid, gallic acid, chlorogenic acid, vanillic acid, resveratrol, anthocyanin, curcumin, salvianolic acid B, bakuchiol, polydatin, eugenol, *p*-coumaric acid, and caffeic acid), and terpenoids (glycyrrhetinic acid, ursolic acid, oleanolic acid, linalool, pinitol, genipin, pinitol, and geraniol) with a total of 30.

Natural products medicine has been practiced to prevent, treat, and cure diseases for thousands of years. Natural products medicine involves using natural compounds, which have relatively complex active ingredients with varying degrees of side effects. Some of these herbal medicines are known to cause nephrotoxicity. Some of the nephrotoxic components from herbs are alkaloids, anthraquinones, flavonoids, and glycosides from natural compounds that cause kidney toxicity [96]. The kidney is the route of excretion of most of the substances present in the natural compounds. The high blood flow rate and sizeable endothelial surface area of the kidneys ensure delivery of large amounts of toxin to the renal parenchyma. High concentrations may be reached in the medulla because of active tubular transport, especially during a state of fluid deprivation. The exact incidence of kidney injury due to nephrotoxic natural compounds is not known.

It is worth noting that inflammatory and antioxidant compounds derived from natural products have therapeutic effects in the AKI-induced model by cisplatin, LPS, sepsis, renal I/R, and hypertension. Further studies are needed to determine the beneficial effects of specific products on humans and other animals with kidney disease to elucidate the detailed mechanisms of their renal protective effects. In addition, while certain natural products are excellent at preventing kidney damage in vitro and in vivo, it is necessary to further researches on the optimal dose to protect against a variety of renal damage.

## 6. Conclusions

AKI is a rapid loss in renal function over a period of hours to days, and a major worldwide health problem. As a result of the decline in renal function, nitrogenous wastes accumulate in the body, resulting in hypernatremia in the blood, and abnormalities in body fluid and electrolyte balance. AKI occurs in about 10% of hospitalized patients and about 30% of patients admitted to the intensive care unit. Despite advances in modern medicine, there are no consistent treatment strategies for preventing the progression to CKD. In this paper, we described the pathogenesis of AKI and the findings of natural products that may potentially assist us in prevention and treatment. So this review summarizes the studies on the effects of three types of natural products on AKI. Studies involving this review have mainly focused on anti-inflammatory and anti-oxidant properties. The causes and clinical features of AKI are very diverse. Hence, studies on natural products with preventive and therapeutic effects related to various causes of AKI should be continuously conducted.

The phytochemicals in medicinal plants have attracted significant attention due to the fewer side effects and being cost-effective. Many compounds such as flavonoids, polyphenols, and terpenoids are effective against induced AKI models. Although natural compounds play an essential role in preventing AKI, it is not yet clear whether these natural compounds can be used as drugs or dietary supplements.

In the future, more research is needed to evaluate the efficacy of plants in AKI prevention, and we expect that this review could be used as a basic paper for meta-analysis, the prevention and treatment of AKI afterward.

## Figures and Tables

**Table 1:** List of some natural products with potential prevention of AKI action.

Name	Model	Prevention/ Treatment	Minimal Active Concentration	Described Effects and Mechanisms	References
Flavonoids					
Quercetin	NRK-52E cells and HK-2 cells	Treatment	10 µM	Reducing the levels of malondialdehyde, lipid ROS and increasing the levels of glutathione	[15]
Luteolin	Cisplatin-induced AKI in mice	Treatment	50 mg/kg	Increased the levels of p53 and its phosphorylation, decreased the levels of PUMA-α, Bax and caspase-3 activity	[16]
Apigenin	Renal ischemia/reperfusion in rats	Prevention	20 mg/kg	Increased the expressions of Bcl-2, p-Akt, PI3K, and down-regulated the expressions of Caspase3 and Bax	[17]
Kaempferol	Cisplatin-induced AKI in mice	Prevention	100 mg/kg	Suppressed levels of TNF-α, iNOS, IL-12, activation of NF-κB, phosphorylation of IκBα and nuclear translocation of p65	[18]
Icariin	Pregnancy-induced hypertension mice	Treatment	100 mg/kg	Improved urinary protein excretion levels and renal tissue damage, upregulation of nephrin expression and downregulation of ANG II	[19]
Myricetin	Cisplatin-induced AKI in mice	Prevention	30 mg/kg	Reduced blood BUN, serum Cr, caspase-3, TNF-α, IL-6, COXI and COXII, MDA levels and, increased GSH level and catalase activity	[20]
Fisetin	LPS-induced septic AKI mice	Treatment	100 mg/kg	Inhibited expression of IL-6, IL-1β, TNF-α, HMGB1, iNOS and COX-2, suppressed of Bcl-2, BAX and cleaved caspase-3	[21]
Galangin	Cisplatin-induced AKI in mice	Prevention	125 mg/kg	Increased SOD, GPx, CAT and GSH levels, inactivated Nrf2, HO-1 and GCLC, inhibitions of ERK and NF-κB signaling pathways	[22]
Tangeretin	Cisplatin-induced AKI in rats	Prevention	8 mg/kg	Reduced MDA, increased GSH, CAT, and SOD activities, elevated Nrf2 expression, downstream effectors IL-1β and TNF-α expression	[23]
Genistein	Renal ischemia/reperfusion in rats	Prevention	15 mg/kg	Increased gene expression levels of TLR4 and TNF-α	[24]
Polyphenols					
Ellagic acid	Cisplatin-induced AKI in mice	Treatment	75 mg/kg	Decreased serum creatinine and reduction of active caspase-3 expression	[25]
Chlorogenic acid	LPS-induced AKI mice	Treatment	30 mg/kg	Inhibiting TLR4/NF-κB signaling pathway, and reduction of active caspase-3	[26]
Gallic acid	Renal ischemia/reperfusion in rats	Prevention	100 mg/kg	Improve the levels of renal MDA, serum GSH, and GPX activity	[27]
Vanillic acid	Cisplatin-induced AKI in rats	Prevention	50 mg/kg	Elevated levels of renal function markers and reduced antioxidant status	[28]
Resveratrol	Cisplatin-induced AKI in rats	Treatment	30 mg/kg	Inhibiting IRE1-NF-κB pathway	[29]
Anthocyanin	Renal ischemia/reperfusion in rats	Prevention	50 mg/Kg	Reduced the elevated levels of IL-1β, IL-6, TNF-α, and MCP-1	[30]
Curcumin	Glycerol-induced AKI in Rats	Prevention/Treatment	200 mg/kg	Inhibiting regulation of the AMPK and Nrf2/HO-1 signaling pathway and ameliorated activating the PI3K/Akt pathway	[31]
Salvianolic Acid B	Cisplatin-induced AKI in rats	Prevention	50 mg/Kg	Activation of the PI3K/Akt/Nrf2 pathway	[32]
Bakuchiol	Sepsis-induced AKI mice	Prevention	45 mg/kg	Blockade of the NF-κB and p38 MAPK signaling pathway	[33]
Polydatin	Sepsis-induced AKI mice	Prevention	30 mg/kg	Blocked by inhibiting SIRT1, and suppressed NLRP3	[34]
Eugenol	Acute pancreatitis rats	Prevention	15 mg/kg	Mild TNF-α activity and low Serum urea and creatinine levels	[35]
p-Coumaric acid	Renal ischemia/reperfusion in rats	Prevention	100 mg/kg	Improved the Cr and BUN levels, reduction in tissue MDA, TNF-α, IL-1β	[36]
Caffeic acid	Cisplatin-induced AKI in rats	Prevention	100 mg/kg	Increase in plasma activities of ALT, AST, ALP, and plasma levels of urea, reduced SOD, CAT, GST and GPx	[37]
Terpenoids					
Glycyrrhetinic acid	Methotrexate-induced nephrotoxicity in rats	Prevention	100 mg/kg	Increase in circulating kidney function markers and TNF-α, up-regulating the Nrf2/ARE signaling	[38]
Ursolic acid	Sepsis-induced AKI mice	Treatment	20 mg/kg	Inhibiting reactive oxygen species, TNF-α, IL-1β, IL-6, and Nf-κB	[39]
Oleanolic acid	Cisplatin-induced AKI in mice	Treatment	40 mg/kg	Inhibiting in caspase-3 and -9 activations and PARP cleavage	[40]
Genipin	LPS-induced AKI in mice	Prevention	15 mg/kg	Increasing the UCP2 content Ameliorating mitochondrial dysfunction, anti-inflammation, and antioxidative activities	[41]
Pinitol	Cisplatin-induced AKI in mice	Treatment	10 mg/kg	Reduction in oxidative stress and cytokines including TNF-α, IL-1β and IL-6.	[42]
Linalool	Cisplatin-induced AKI in rats	Prevention	50 mg/kg	Managed oxidation systems of Nrf2-mediated pathway and diminished TNF-α, IL-1β, IL-6, and NF-κB	[43]
Geraniol	Cisplatin-induced AKI in rats	Treatment	100 mg/kg	Abrogated oxidative stress and downregulated the MAPK, STAT-1, p53, p21 and MMP9	[44]

Abbreviation: AKI; Acute kidney injury, LPS; Lipopolysaccharides.

## Data Availability

Not applicable.
